# FIT: statistical modeling tool for transcriptome dynamics under fluctuating field conditions

**DOI:** 10.1093/bioinformatics/btx049

**Published:** 2017-01-31

**Authors:** Koji Iwayama, Yuri Aisaka, Natsumaro Kutsuna, Atsushi J Nagano

**Affiliations:** 1 Research Institute for Food and Agriculture, Ryukoku University, Otsu, Shiga, Japan; 2 LPixel Inc, Hongo, Bunkyo-ku, Tokyo, Japan; 3 Department of Integrated Biosciences, Graduate School of Frontier Sciences, The University of Tokyo, Kashiwa-shi, Chiba, Japan; 4 Department of Plant Life Science, Faculty of Agriculture, Ryukoku University, Otsu, Shiga, Japan; 5 Center for Ecological Research, Kyoto University, Otsu, Shiga, Japan; 6 JST PRESTO, Kawaguchi, Saitama, Japan

## Abstract

**Motivation:**

Considerable attention has been given to the quantification of environmental effects on organisms. In natural conditions, environmental factors are continuously changing in a complex manner. To reveal the effects of such environmental variations on organisms, transcriptome data in field environments have been collected and analyzed. Nagano *et al.* proposed a model that describes the relationship between transcriptomic variation and environmental conditions and demonstrated the capability to predict transcriptome variation in rice plants. However, the computational cost of parameter optimization has prevented its wide application.

**Results:**

We propose a new statistical model and efficient parameter optimization based on the previous study. We developed and released FIT, an R package that offers functions for parameter optimization and transcriptome prediction. The proposed method achieves comparable or better prediction performance within a shorter computational time than the previous method. The package will facilitate the study of the environmental effects on transcriptomic variation in field conditions.

**Availability and Implementation:**

Freely available from CRAN (https://cran.r-project.org/web/packages/FIT/).

**Supplementary information:**

Supplementary data are available at *Bioinformatics* online

## 1 Introduction

Variation in environmental factors affects various aspects of organisms. The comprehensive quantification of environmental effects on organisms is an emerging problem. For example, gene-environment interactions have been studied to explain the heritability of complex diseases that could not be explained by conventional genome-wide association studies (Thomas, 2010). In addition, concerns about organisms’ adaptation and response to changes in environmental conditions has been growing because of climate change (Ahuja *et al.*, 2010; Hoffmann and Sgrò, 2011; Nicotra *et al.*, 2010; Weston *et al.*, 2008; Xu, 2016).

There is a trade-off between precise control of and minimal intervention to the environment (Jones, 2013). Any results obtained in the field more accurately reflect a plant’s environmental response in natural conditions. However, these results are difficult to interpret, because environmental factors are continuously changing in a complex manner and exhibit diurnal oscillations, seasonal changes, and long-term trends. On the other hand, experiments conducted in controlled conditions provide results that are more precise but not necessarily reflective of the plant’s behavior in natural conditions. For example, photosynthetic responses to fluctuating, environments like natural conditions differ from those to controlled, constant environmental conditions (Yamori, 2016). At the molecular level, traits observed in laboratory conditions are not always consistent with those observed in natural conditions (Malmberg *et al.*, 2005; Mishra *et al.*, 2012; Weinig *et al.*, 2002). Further, even if similar physiological plasticity is observed, the molecular responses of plants to drought stress can vary between the controlled conditions of greenhouses and the uncontrolled conditions of the field (Lovell *et al.*, 2016).

To reveal the effects of such fluctuating environments, transcriptome data in field environments have been collected (Alvarez *et al.*, 2015). In particular, the transcriptomic variation of plants in fields has been studied (Hayes *et al.*, 2010; Izawa *et al.*, 2011; Nagano *et al.*, 2012; Plessis *et al.*, 2015; Sato *et al.*, 2011; Richards *et al.*, 2012). To bridge the gap between natural and laboratory conditions, Nagano *et al.* (2012) proposed a model to relate the transcriptomic variation of plants in a field to environmental conditions and applied it to large-scale transcriptome data of samples collected in a field. They demonstrated that the model can precisely predict transcriptome variations using meteorological data. However, the vast computational cost required to optimize parameters and select the best model has restricted the use of the model.

To accelerate transcriptomic studies in fields, we propose a new statistical model based on that proposed by Nagano *et al.* (2012). The previous model contains some distinct functions representing diurnal changes in sensitivity and the characteristics of responses to environmental stimuli. The new model reduces the computational cost by unifying these functions. We also propose a cluster-based approach for optimizing parameters, in which we reuse the optimization result of one gene for other genes in the same cluster. This approach significantly reduces the cost of searching for the optimum parameter value. Both the previous and new models are intended for application to microarray data; however, the use of RNA-Seq (Wang *et al.*, 2009) technology is rapidly increasing. To apply our model to RNA-Seq data, we incorporated precision weights for normalized log-counts into the model as the voom method (Law *et al.*, 2014). In addition, we developed and released FIT, an R package that provides efficient parameter optimization, model selection, and transcriptome prediction of unsequenced samples. This is the first tool to integrate the transcriptome data from field samples and meteorological data by modeling their relation.

## 2 Methods

### 2.1 Previous model

Before explaining our new model, we briefly review the previous plant transcriptome model (Nagano *et al.*, 2012). Let *N* be the number of samples and $s^{\left(i\right)}$ be an *N*-element vector, where each element is the $\;{\text{log}}_{2}$-transformed value of the normalized expressions for gene *i* in each sample. In the model, $s^{\left(i\right)}$ is described as

(1) $$s^{\left(i\right)}=X^{\left(i\right)}\beta^{\left(i\right)}+\varepsilon^{\left(i\right)},$$

where $X^{\left(i\right)}$ and $\beta^{\left(i\right)}$ are an N×7 design matrix and regression coefficients, respectively. The independently and identically distributed noise $\varepsilon^{\left(i\right)}$ is drawn from a Gaussian distribution. The design matrix $X^{\left(i\right)}$ is constructed as

(2) $$X^{\left(i\right)}=\left(1,d,c^{\left(i\right)},r^{\left(i\right)},d○c^{\left(i\right)},d○r^{\left(i\right)},n\right).$$

Here, **1** is a vector in which all elements are 1 and $d,\;c^{\left(i\right)},\;r^{\left(i\right)}$, and n are values designating the plant’s age, circadian clock, response to environmental stimuli, and genotype, respectively. The element-wise products $d○c^{\left(i\right)}$ and $d○r^{\left(i\right)}$ are the interactions between the age and the clock and the age and the environmental response, respectively. The plant’s age d is a vector of the numbers of days after transplanting scaled to have a range 1 and a mean 0. In the previous study, the authors used two rice cultivars (Nipponbare and Norin 8) as samples. If the genotype of sample *j* is Norin 8, the *j*-th element of the genotype n is set to 1; otherwise, to 0.

Whereas d and n are independent of the genes, the circadian clock and the response to environmental stimuli are gene specific. Hence, we place a superscript (*i*), e.g. $c^{\left(i\right)}$ and $r^{\left(i\right)}$. The circadian clock on gene *i* in sample *j* is described by a cosine curve with a 24 h period and the gene specific phase $\varphi^{\left(i\right)}$. It is defined as

(3) $$c_{j}^{\left(i\right)}=\frac{\;\text{cos}\;\left(2\pi (t_{j}-\varphi^{\left(i\right)})/24\right)}{2},$$

where *t_j_* is the time when sample *j* was obtained.

The response to environmental stimuli of gene *i* in sample *j* takes the form

(4) $$r_{j}^{\left(i\right)}=\frac{\sum_{T=t_{j}-p^{\left(i\right)}}^{t_{j}}g(T)f(w_{T}-\theta^{\left(i\right)})}{a^{\left(i\right)}}-b^{\left(i\right)},$$

where *g*(*T*) is a gate aperture used to explain time-of-day-specific responses to an environmental stimulus, $p^{\left(i\right)}$ is the time period during which gene *i* was affected by an environmental stimulus, *w_T_* is the value of a meteorological parameter at time *T*, $\theta^{\left(i\right)}$ is the response threshold to the stimulus, and *f*(*x*) determines the response to the environmental stimulus. $r^{\left(i\right)}$ is scaled by factors $a^{\left(i\right)}$ and $b^{\left(i\right)}$ as the plant’s age. For *f*(*x*), two types of response model are considered: dose-dependent (dd) and dose-independent (di). In the dd model, the response function is defined as

(5) $$f_{dd,p}(x)=\max(0,x).$$

Similarly, the function in the di model is defined as

(6) $$f_{di,p}(x)=\{\begin{array}{cc} 1, & x>0,\\ 0, & \text{otherwise}. \end{array}$$

These two response model types assume that a plant responds to an environmental stimulus over the threshold. Two other types can also be considered, that is, a plant responds to a stimulus under the threshold, such as

(7) $$\begin{array}{c} f_{dd,n}(x)=\max(0,-x),\\ f_{di,n}(x)=\{\begin{array}{cc} 1, & x<0,\\ 0, & \text{otherwise}. \end{array} \end{array}$$

There are three types of gate function *g*(*T*). The first model is a no-gate model, which takes 1 for any *T*. In the second model, the gate function is defined as

(8) $$g_{\;\text{cos}\;}(T)=\frac{\;\text{cos}\;\left(2\pi \left(T-\psi \right)/24\right)+1}{2}.$$

The third model assumes that the gate fully opens for a specific duration and completely closes at other times, i.e.,

(9) $$g_{\text{rect}}(T)=\{\begin{array}{cc} 1, & o^{\left(i\right)}<T<(o^{\left(i\right)}+l^{\left(i\right)}),\\ 0, & \text{otherwise}. \end{array}$$

Here, $o^{\left(i\right)}$ is the opening time of the gate and $l^{\left(i\right)}$ is the opening length.

Parameters are optimized to maximize the likelihood by using the Nelder–Mead algorithm (Nelder and Mead, 1965). Because the likelihood function has multiple local maxima, a grid search is performed before the optimization. Regression coefficients $\beta^{\left(i\right)}$ and the phase of the circadian clock $\varphi^{\left(i\right)}$ are optimized using the nonlinear least-squares method and the likelihood is calculated on each grid point of the remaining parameters. The optimization by the Nelder–Mead algorithm starts from the parameter values of the grid point with the largest likelihood. The response function to environmental stimuli with the largest likelihood is selected from four response model types ($f_{dd,p}(x),\;f_{di,p}(x),\;f_{dd_{n}}(x)$, and $f_{di_{n}}(x)$). Because different gate function types have different degrees of freedom, the optimum gate function type is selected by performing likelihood-ratio tests. It can be considered that some variables in Equation (1) may contribute to neither the explanation nor the prediction for the expression patterns of many genes. Thus, a parameter reduction process that repeats parameter optimizations and likelihood-ratio tests is performed to obtain the simplest models.

### 2.2 New model

Although the previous model achieved a detailed description of transcriptome fluctuations in complex environments, the parameter optimization for all genes is computationally expensive. The computational cost of the optimization is due mainly to the existence of different model types, i.e., different types of response models and gate functions, and variable selection. We need to optimize and compare each model repeatedly. Therefore, to reduce computational cost, we unified the different model types.

To unify the *dd* and *di* responses to environmental stimuli, we redefined the response model, when a plant responds to an environmental stimulus over the threshold, as

(10) $${\tilde{f}}_{p}(x)=\max\left(0,\tanh\left(\text{exp}\;\left(\gamma_{f}\right)x\right)\right)\sqrt{\;\text{exp}\;\left(-2\gamma_{f}\right)+1}.$$

**Fig. 1:**
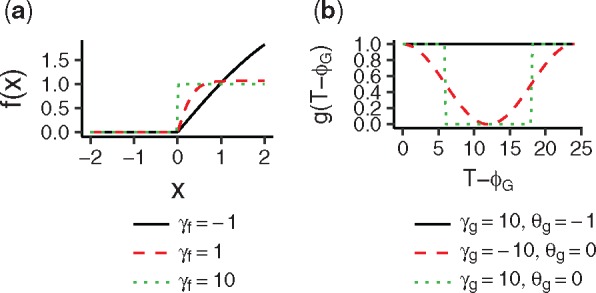
Response functions (**a**) and gate functions (**b**) for various parameter values

As *γ_f_* approaches minus infinity, this function approaches the *dd* response function. Conversely, this function approaches the *di* response function in the limit of $\gamma_{f}\to \infty$ (Fig. 1(a)). Here, $\sqrt{\;\text{exp}\;(-2\gamma_{f})+1}$ ensures that the outputs of this function have almost constant scales regardless of the value of *γ_f_*. When a plant responds to a stimulus under the threshold, the dd and di responses are unified as

(11) $${\tilde{f}}_{n}(x)=\max\left(0,\tanh\;\left(-\text{exp}\;\left(\gamma_{f}\right)x\right)\right)\sqrt{\;\text{exp}\;\left(-2\gamma_{f}\right)+1}.$$

We also defined the new gate function as

$$h(C)=\tanh\;\left(\text{exp}\;\left(\gamma_{g}\right)\left(C-\theta_{g}\right)\right),$$

(12) $$\tilde{g}(T)=\frac{h\;\left(\text{cos}\;\left(2\pi \left(T-\psi \right)/24\right)\right)-h(-1)}{h(1)-h(-1)},$$

where *γ_g_* controls the shape of the gate as *γ_f_* in Equations (10) and (11), and *θ_g_* determines the opening length. When the value of *θ_g_* is smaller than –1, this function corresponds to the no-gate model in the limit $\gamma_{g}\to \infty$. The gate function in Equation (8) is approximated by this function with a smaller value of *γ_g_* and that in Equation (9) is approximated with a larger value of *γ_g_* [Fig. 1(b)].

To eliminate the need to optimize the phase of the circadian clock $\phi^{\left(i\right)}$ by the nonlinear least-squares method, we represent the circadian clock by the weighted sum of sine and cosine curves instead of one cosine curve (Equation 3). The weights of these sine and cosine curves are selected by linear regression together with other regression coefficients. Let $\beta_{\;\text{sin}\;}^{\left(i\right)}$ and $\beta_{\;\text{cos}\;}^{\left(i\right)}$ be the regression coefficients of the sine and cosine components of the clock. Using these coefficients, the clock phase is obtained by

(13) $$\varphi^{\left(i\right)}=\arg(\beta_{\;\text{cos}\;}^{\left(i\right)}+i\beta_{\;\text{sin}\;}^{\left(i\right)}).$$

Similarly, the interactions between the plant’s age and the circadian clock are also represented as the weighted sum of the interactions between the age and cosine and sine curves, and its phase is obtained by these regression coefficients.

As in the previous model, the parameters of the new model are optimized through two steps: the determination of the initial value by a grid search, and nonlinear optimization by the Nelder–Mead algorithm. In the first step, we optimize the parameters related to environmental responses, which are $p^{\left(i\right)},\;\theta^{\left(i\right)}$, *γ_f_*, *γ_g_*, and *θ_g_*, and the response function (${\tilde{f}}_{p}$ or ${\tilde{f}}_{n}$) by a grid search. The remaining parameter $\beta^{\left(i\right)}$ is optimized by linear regression for each setting and the setting that achieves the lowest mean squared error is searched. In the second step, parameters other than $\beta^{\left(i\right)}$ are optimized by the Nelder–Mead algorithm. After the optimization of the environmental response parameters, we select variables simultaneously with the optimization of the regression coefficients $\beta^{\left(i\right)}$ using an L1 regularization instead of repeating log-likelihood tests. To select sine and cosine curves representing the circadian clock (and the interaction between the age and the circadian clock) into or out of a model together, we used group lasso (Yuan and Lin, 2006) rather than lasso (Tibshirani, 1996). By using group lasso, sparse (i.e. a small number of non-zero) coefficients can be obtained. Let ${\hat{s}}_{j}^{\left(i\right)}$ be the predicted value of the expression of gene *i* in sample *j*. The cost function to be minimized is defined as

$$L(\beta )=\sum_{j=1}^{N}{\left({\hat{s}}_{j}^{\left(i\right)}-s_{j}^{\left(i\right)}\right)}^{2}$$

(14) $$+\lambda \left(\sum_{k\in I}\zeta_{k}|\beta_{k}|+\zeta_{c}\sqrt{\beta_{\;\text{cos}\;}^{2}+\beta_{\;\text{sin}\;}^{2}}+\zeta_{dc}\sqrt{\beta_{d\;\text{cos}\;}^{2}+\beta_{d\;\text{sin}\;}^{2}}\right).$$

Here, I={d,r,dr,n} is the index set of the regression coefficients of the plant’s age, the response to environmental stimuli, the interaction between the age and the response, and the genotype. In this equation, the first term is the sum of the squared residuals and the second term is the regularization term. With a larger value of *λ*, we obtain fewer non-zero coefficients. To achieve consistent variable selection, we use adaptive weights *ζ_j_* for penalizing different covariates as in the adaptive lasso (Zou, 2006) or the adaptive group lasso (Wang and Leng, 2008). Let $\tilde{\beta }$ denote the coefficients obtained by ordinary least squares regression. The adaptive weights are defined as $\zeta_{k}=1/{\tilde{\beta }}_{k}^{2}\;(k\in \{d,n\})$, $\zeta_{k}=7/{\tilde{\beta }}_{k}^{2}\;(k\in \{r,dr\}),\;\zeta_{c}=1/({\tilde{\beta }}_{\;\text{cos}\;}^{2}+{\tilde{\beta }}_{\;\text{sin}\;}^{2})$, and $\zeta_{dc}=1/({\tilde{\beta }}_{d\;\text{cos}\;}^{2}+{\tilde{\beta }}_{d\;\text{sin}\;}^{2})$. Because the responses to environmental stimuli contain seven parameters, the adaptive weights for the response and the interaction between the response and the age are multiplied by seven.

The optimum value of *λ* is selected by cross validation (CV) for each gene. We chose the largest value of *λ* for which the CV error was smaller than the sum of the minimum CV error and the standard error of CV errors. Because CV incurs high computational costs, we used the approximation of leave-one-out CV errors (Obuchi and Kabashima, 2016). A leave-one-out CV error and its standard error is approximated by the mean and the standard deviation of

(15) $${\left(1+\sum_{k,l}X_{jk}^{\left(i\right)}X_{jl}^{\left(i\right)}\chi_{kl}^{\left(ij\right)}\right)}^{2}{\left({\hat{s}}_{j}^{\left(i\right)}-s_{j}^{\left(i\right)}\right)}^{2},$$

over all samples *j*. Here, $\chi^{\left(ij\right)}$ is the matrix given by

$$\chi^{\left(ij\right)}={\left({\tilde{X}}^{(i)T}{\tilde{X}}^{\left(i\right)}\right)}^{-1}$$

$$+\frac{{\left({\tilde{X}}^{(i)T}{\tilde{X}}^{\left(i\right)}\right)}^{-1}{\tilde{X}}_{j\cdot }^{(i)T}{\tilde{X}}_{j\cdot }^{\left(i\right)}{\left({\tilde{X}}^{(i)T}{\tilde{X}}^{\left(i\right)}\right)}^{-1}}{1-{\tilde{X}}_{j\cdot }^{\left(i\right)}{\left({\tilde{X}}^{(i)T}{\tilde{X}}^{\left(i\right)}\right)}^{-1}{\tilde{X}}_{j\cdot }^{(i)T}},$$

where ${\tilde{X}}^{\left(i\right)}$ and ${\tilde{X}}_{j\cdot }^{\left(i\right)}$ denote the submatrix of $X^{\left(i\right)}$ corresponding to the set of covariates with non-zero regression coefficients, and its *j*-th row. Although this approximation is based on the assumption that the numbers of samples and covariates are sufficiently large, we confirmed that it is sufficiently accurate even for our model, which contains only several covariates.

### 2.3 Cluster-based optimization for computational time reduction

For further computational reduction, we omit the grid search (which is the most time-consuming step in the optimization) for almost all genes. Similar models would be optimum for genes exhibiting similar expression patterns. Hence, it is expected that the optimum model for one representative gene of a cluster can be used as the initial value of the Nelder–Mead optimization for genes, the expression pattern of which is similar to the pattern of the representative gene of the cluster.

Before optimization, we perform clustering of expression patterns using the affinity-propagation method (Frey and Dueck, 2007), which automatically provides an appropriate number of clusters and their exemplars. For each cluster, we optimize the parameter values for the exemplar of the cluster using the procedure mentioned above, and use the optimized value as the initial value for the Nelder–Mead method for other genes in the cluster.

### 2.4 Application to RNA-seq data

Recently, RNA-Seq (Wang *et al.*, 2009) has become a widely used technology to quantify transcriptomes. While we employed the lognormal distribution for microarray data, RNA-Seq data are discrete in nature and modeled by a negative binomial distribution (Robinson *et al.*, 2010). We associated a precision weight with each individual normalized observation as in the voom method (Law *et al.*, 2014), which allows us to apply methods developed for microarray data to RNA-Seq data. Precision weights are estimated based on the mean-variance relationship of the data according to the following procedure. The log-count per million (log-cpm) value for each read count is defined as

(16) $$y_{j}^{\left(i\right)}={\text{log}}_{2}\left(\frac{r_{j}^{\left(i\right)}+0.5}{R_{j}+1.0}\times 10^{6}\right),$$

where $r_{j}^{\left(i\right)}$ denotes the read count of gene *i* for RNA sample *j* and $R_{j}=\sum r_{j}^{\left(i\right)}$ is the total number of reads for sample *j*. For each gene, a model is fitted to the log-cpm value $y_{j}^{\left(i\right)}$ and its residual standard deviation $\sigma^{\left(i\right)}$ is obtained. In this study, we smoothed the time series of the log-cpm values and calculated residual standard deviations. Let ${\bar{r}}^{\left(i\right)}$ denote the average log-count value defined as

(17) $${\bar{r}}^{\left(i\right)}={\bar{y}}^{\left(i\right)}+{\text{log}}_{2}(\bar{R})-{\text{log}}_{2}(10^{6}).$$

Here, ${\bar{y}}^{\left(i\right)}$ and $\bar{R}$ are the average log-cpm value and the geometric mean of the total number of reads plus one, respectively. Similarly, each smoothed log-cpm value of gene *g* for sample *i* is converted to the smoothed log-count ${\bar{\lambda }}_{j}^{\left(i\right)}$. Fitting the LOWESS curve (Cleveland, 1979) to square-root standard deviations $\sqrt{\sigma^{\left(i\right)}}$ as a function of mean log-counts ${\bar{r}}^{\left(i\right)}$, a piecewise linear function lo() is yielded. Then, the predicted square-root standard deviation of $y_{j}^{\left(i\right)}$ is $lo({\bar{\lambda }}_{j}^{\left(i\right)})$ and the voom precision weight is defined as

(18) $$w_{j}^{\left(i\right)}=\frac{1}{lo{\left({\bar{\lambda }}_{j}^{\left(i\right)}\right)}^{4}}.$$

Precision weights are incorporated into the model by replacing the first term in Equation (14) with the weighted sum of squared residuals.

## 3 Datasets

### 3.1 Meteorological data

We used meteorological data measured every 60 s at a meteorological station (Tsukuba (Tateno), 36°03′N, 140°08′E, attitude 25.2m) of the Japan Meteorological Agency. The data consists of wind intensity = (m/s), air temperature (°C), relative humidity (%), atmospheric pressure (hPa), precipitation (mm), and global radiation (${\text{kJm}}^{-2}{\text{min}}^{-1}$).

### 3.2 Synthetic gene expression data

In order to confirm that correct models can be selected by the proposed method, we synthetically generated RNA-Seq data assuming the following situation. Rice plants were transplanted into a paddy field on June 1. Samples were collected every week from June 12 to September 18, 2008 for 24 h. In order to verify influence of sampling design on model selection and parameter optimization, five types of sampling were considered: one sample at each time at intervals of 2 h, two samples at each time at intervals of 4 h, three samples at each time at intervals of 6 h, four samples at each time at intervals of 8 h, and six samples at each time at intervals of 12 h. The total number of samples for each type was 180. For evaluation of the predictive capability, we also computed gene expressions of samples assumed to be collected every week from June 12 to September 18, 2009 for 24 h at intervals of 2 h.

The read count of gene *i* for RNA sample *j* was generated from a negative binomial distribution:

(19) $$p(r_{j}^{\left(i\right)})=\frac{\Gamma (\phi^{-1}+r_{j}^{\left(i\right)})}{r_{j}^{\left(i\right)}!\Gamma (\phi^{-1})}{\left(\frac{1}{1+{\left(\mu_{j}^{\left(i\right)}\phi_{i}\right)}^{-1}}\right)}^{r_{j}^{\left(i\right)}}{\left(\frac{1}{1+\mu_{j}^{\left(i\right)}\phi_{i}}\right)}^{\phi_{i}^{-1}},$$

where $\mu_{j}^{\left(i\right)}$ and $\phi_{i}$ are the mean and the dispersion parameter, respectively. The mean values varied according to the circadian clock, the response to environmental stimuli, and the plant’s age at the times of sampling, according to the 31 model equations shown in Supplementary Table S1. In these equations, the logarithm of the average expression value for each gene *α_j_* followed a normal distribution with an average of 5 and a standard deviation of 1.

To decide dispersion parameters, we estimated the mean-dispersion trend from the Pickrell real RNA-Seq data set (Pickrell *et al.*, 2010), which is available from the tweeDESeqCountData R package (http://www.creal.cat/jrgonzalez/software.htm), using the method implemented in the edgeR package (Zhou *et al.*, 2014). From the estimated trend, we decided the value of the dispersion parameter corresponding to the average expression value for each gene *α_j_*. Because we use the log-count per million values for analysis, 9969 constantly expressed genes ($\mu_{j}^{\left(i\right)}=\alpha_{i},\;\forall j$) were also considered in order to suppress the influence of the variation of the total read counts. The dispersion parameters of these constantly expressed genes were also decided according to the estimated mean-dispersion trend.

### 3.3 Real gene expression data

We also analyzed the same data as Nagano *et al.* (2012), which consisted of microarray data from 461 samples of mature leaves of rice plants in a paddy field at Tsukuba collected in 2008, and 108 samples collected in 2009. We used data from the samples collected in 2008 for model selection and parameter fitting, and those from 2009 for evaluation of the model’s predictive capability. The samples collected in 2008 can be categorized into six groups: 24 h of samples collected at intervals of 2 h starting at 7:00 am on August 12, 2008 (8 samples at each time × 13 time points =104 samples); 48 h of samples collected at intervals of 2 h starting at 10:00 am on June 5, June 19, July 3, July 17, August 7, August 14, August 21, August 28, and September 11, 2008 (225 samples); samples collected at noon every other week from June 3 to September 23, 2008 (three samples at each time × 17 time points =51 samples); samples collected at 12:00 am every other week from June 4 to September 24, 2008 (2 samples at each time × 17 time points =34 samples); samples collected from 5:00 pm to 8:00 pm at intervals of 10 min on August 7, 2008 (19 samples); samples collected from 3:50 am to 6:00 am at intervals of 10 min on August 8, 2008 (two samples at each time × 14 time points =28 samples). The samples collected in 2009 can be categorized into six groups: 48 h of samples collected at intervals of 6 h starting at noon on August 10, 2009 (two samples at each time × 9 time points =18 samples); 24 h of samples collected at intervals of 2 h starting at 7:00 am on August 24, 2009 (6 samples at each time × 13 time points =78 samples); two samples collected at noon on August 31, 2009; two samples collected at 6:00 pm on August 31, 2009; four samples collected at 11:00 am on October 8, 2009; four samples collected at 11:00 am on October 9, 2009. This information is also summarized in Supplementary Tables S2 and S3. We extracted 17 616 genes having *log*_2_-transformed signals larger than 5 in 80% of the samples from 2008.

To validate the proposed model’s applicability to RNA-Seq data, we generated pseudo RNA-Seq data from the same microarray data. The pseudo RNA-Seq data of sample *j* were sampled from the multinomial distribution:

(20) $$p\left({\left\{r_{j}^{\left(i\right)}\right\}}_{i}\right)=\frac{R_{j}!}{\prod_{i}r_{j}^{\left(i\right)}}{\left(\frac{\prod_{i}2^{s_{j}^{\left(i\right)}}}{\sum_{k}2^{s_{j}^{\left(k\right)}}}\right)}^{r_{J}^{\left(i\right)}}.$$

Here, $R_{i}=10^{8}$ is the total number of reads.

## 4 Results

### 4.1 Synthetic gene expression data

We used synthetic RNA-Seq data collected in 2008 for parameter optimization and model selection; we used synthetic RNA-Seq data collected in 2009 to evaluate the prediction capabilities of the optimized model. Mean squared errors (MSE) and correlation coefficients between the predicted and synthetic gene expressions are plotted in Supplementary Figures S1 and S2. These figures indicate that sampling at intervals of 4 h provides better predictive performance than sampling at longer intervals. It can be considered that sampling at 4 h effectively captures diurnal variation of gene expression patterns. Although the predictive performance of sampling at 2 h intervals was comparable to that of sampling at 4 h intervals, longer intervals are preferable in terms of sampling labor. Hence, only results of sampling at 4 h intervals are shown below.

The selected models for variably expressed genes are summarized in Supplementary Table S4. This results indicate that the correct models were selected for 29 out of 31 genes. It is important to note that absolute values of coefficients do not always correspond to those of the true models in Supplementary Table S1, because of the normalization and nonlinear transformation of inputs. Further, the constant model, where all coefficients are zero, was correctly selected for 8441 out of 9969 constantly expressed genes. These results indicate that the variable selection through L1 regularization worked well.

We also confirmed that the optimized parameter values were consistent with those of the true models for variably expressed genes. The polar plot of the optimized coefficients of circadian clocks $\beta_{\;\text{cos}\;}^{\left(i\right)}+i\beta_{\;\text{sin}\;}^{\left(i\right)}$ is shown in Supplementary Figure S3. The phases of circadian clocks $\varphi^{\left(i\right)}=\arg(\beta_{\;\text{cos}\;}^{\left(i\right)}+i\beta_{\;\text{sin}\;}^{\left(i\right)})$ clearly correspond to those of the true models. The optimized memory time periods were sharply distributed around the values, which were very close to the true values (Supplementary Fig. S4). To confirm whether the optimized parameter values of responses to environmental stimuli were consistent with those of the true models, we computed the responses as follows:

$${\tilde{f}}^{\left(i\right)}(T)=\beta_{r}\max(0,\tanh(\rho^{\left(i\right)}\;\text{exp}\;(\gamma_{f}^{\left(i\right)})(T-\theta^{\left(i\right)})))$$

(21) $$\times \sqrt{\;\text{exp}\;(-2\gamma_{f}^{\left(i\right)})+1}.$$

Here, *T* denotes temperature, and $\rho^{\left(i\right)}$ indicates the type of response, that is, $\rho^{\left(i\right)}=1$ if a plant responds to temperatures over the threshold $\theta^{\left(i\right)}$, otherwise, $\rho^{\left(i\right)}=-1$. The optimized responses of variably expressed genes are plotted in Supplementary Figure S5. The optimized models of dose-independent genes tend to exhibit step function-like responses compared to those of dose-dependent genes. Although some dose-independent genes with positive (negative) coefficients of responses respond to temperatures over (under) the threshold contrary to the true models, they can match the response functions by adding (subtracting) certain constant values.

### 4.2 Real gene expression data

We optimized the parameters of the model using the microarray data of samples collected in 2008 by normal optimization, in which we performed both the grid search and the nonlinear optimization for all genes, and cluster-based optimization (section 2.3). We also applied cluster-based optimization to pseudo RNA-Seq data generated from the same microarray data. We performed all optimizations on an Amazon EC2 m3.medium instance (one Intel Xeon E5-2670 v2 processor, 3.75 GiB memory). The normal optimization took 81.4 s per gene on average. If we performed the normal optimization using a single m3.medium instance, we could optimize parameters for 17 616 genes within 17 days. The affinity-propagation method yielded 500 clusters and their exemplars. After normal optimization for these exemplars, we performed nonlinear optimization for other genes using the Nelder–Mead method. The nonlinear optimization took 22.3 s per gene. We could optimize parameters for 17 616 genes within 5 days by using a single instance. In the previous study (Nagano *et al.*, 2012), the parameter optimization took about 30 days using a high-performance cluster computer. Even after taking into consideration advances in computer technology, the new model improved computational efficiency.

**Fig. 2:**
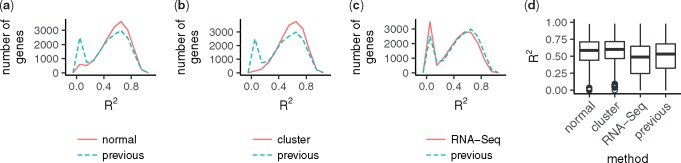
Comparison of coefficients of determination (*R*^2^). Distributions of *R*^2^ from the previous model are compared to those of the new model with normal optimization (**a:** "normal"), cluster-based optimization (**b:** "cluster"), and cluster-based optimization with pseudo RNA-Seq data (**c:** "RNA-Seq"). (d) Boxplot of *R*^2^ values

The fitness of the new model was compared to that of the previous model by coefficients of determination *R*^2^, defined as

(22) $$R^{2}=1-\frac{\sum_{j}{\left(s_{j}^{\left(i\right)}-{\hat{s}}_{j}^{\left(i\right)}\right)}^{2}}{\sum_{j}{\left(s_{j}^{\left(i\right)}-{\bar{s}}^{\left(i\right)}\right)}^{2}}$$

for microarray data and

(23) $$R^{2}=1-\frac{\sum_{j}{\left(y_{j}^{\left(i\right)}-{\hat{y}}_{j}^{\left(i\right)}\right)}^{2}}{\sum_{j}{\left(y_{j}^{\left(i\right)}-{\bar{y}}^{\left(i\right)}\right)}^{2}}$$

for pseudo RNA-Seq data. Here, ${\hat{s}}_{j}^{\left(i\right)}$ and ${\hat{y}}_{j}^{\left(i\right)}$ are the predictions of models for microarray and pseudo RNA-Seq data, and ${\bar{s}}^{\left(i\right)}$ and ${\bar{y}}^{\left(i\right)}$ denote the average *log*_2_-transformed signals and pseudo log-rpm values, respectively. Figure 2 shows the *R*^2^ values. The *R*^2^ values of three different optimizations were comparable to those of the previous model.

**Fig. 3:**
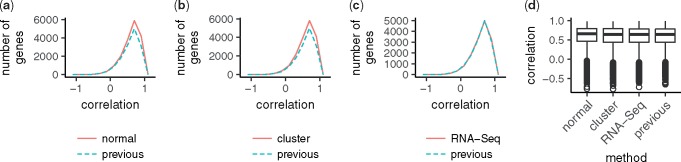
Comparison of gene-wise correlation across samples. Each panel is illustrated as in Fig. 2. The null model, in which the expression is the constant, was selected for some genes in the previous study. Because correlation coefficients for such genes cannot be defined, the total number of genes contained in the plots of the previous study (dashed) is smaller than that of the new model (solid)

**Fig. 4:**
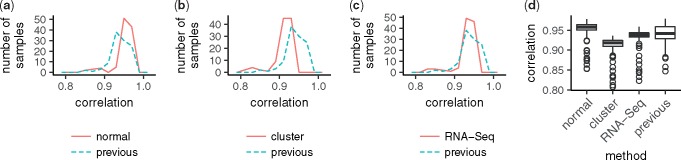
Comparison of sample-wise correlation across genes. Each panel is illustrated as in Figure 2

To assess the new model’s capability to predict gene expression, we predicted the microarray data or pseudo RNA-Seq data of samples collected in 2009. We compared gene-wise mean squared errors across samples. The mean squared errors of the new model with normal optimization, cluster-based optimization, and cluster-based optimization using pseudo RNA-Seq data were smaller than those of the previous model for 12 125, 12 209 and 9305 out of 17 616 genes, respectively. Hence, the new model yields better predictions than the previous model, regardless of whether the parameters are optimized by the normal or the cluster-based methods and whether the data are measured using a microarray or RNA-Seq. We also calculated gene-wise correlation across samples and sample-wise correlation across genes between prediction and observation. The gene-wise correlation is illustrated in Figure 3. The distributions of the correlation coefficients of the new model were comparable to that of the previous model, regardless of the methods of measurement and parameter optimization. Further, the sample-wise correlations of all three tests were higher than 0.9 for most samples and improved in comparison to those of the previous model (Fig. 4).

Next, we compared the models selected by the normal optimization to those obtained in the previous study (Nagano *et al.*, 2012). Table 1 shows the numbers of genes for which each environmental factor was selected in the previous study and the normal optimization of the new model. Each row and column shows the number of genes for which the corresponding environmental factor was selected in the previous study and the normal optimization of the new model, respectively. The bottom row represents the model without the environmental effects. In the normal optimization, if the weight for the environmental effects is zero, the gene is counted as "new none." The bold-faced numbers in the diagonal cells of the table indicate the numbers of genes for which the same environmental factor was selected in the previous study and the normal optimization of the new model. The same environmental factor was selected in both models for 4523 (sum of the first six diagonal cells) out of 7496 genes, for which the selected models contain the response to any environmental factors in both the previous and new studies. Meanwhile, in the previous study, the model without the environmental effects was selected for 10 120 genes (sum of the bottom row); it was selected by the normal optimization of the new model for only 5714 genes (sum of the rightmost column). The previous model tends to select the model without the environmental effects about twice as frequently as the new model. This difference is probably due to differences in the model selection, i.e., likelihood ratio tests and group lasso.

**Table 1:** Comparison of selected models

	New wind	New temperature	New humidity	New atmosphere	New radiation	New precipitation	New none
Previous wind	**56**	20	8	3	7	4	113
Previous temperature	36	**3762**	99	68	92	15	877
Previous humidity	10	49	**340**	7	27	14	237
Previous atmosphere	6	18	6	**38**	7	6	78
Previous radiation	26	238	91	49	**309**	20	647
Previous precipitation	4	8	13	5	10	**18**	55
Previous none	233	4434	641	343	643	119	**3707**

**Fig. 5:**
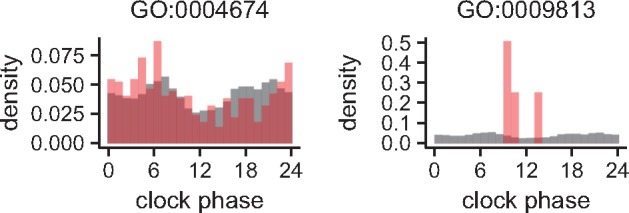
Distributions of clock phases φ of genes annotated with protein serine/threonine kinase activity (GO:0004674) and flavonoid biosynthetic process (GO:0009813). Fractions of genes annotated with the term and not annotated with the term are shown in red and gray, respectively

**Fig. 6:**
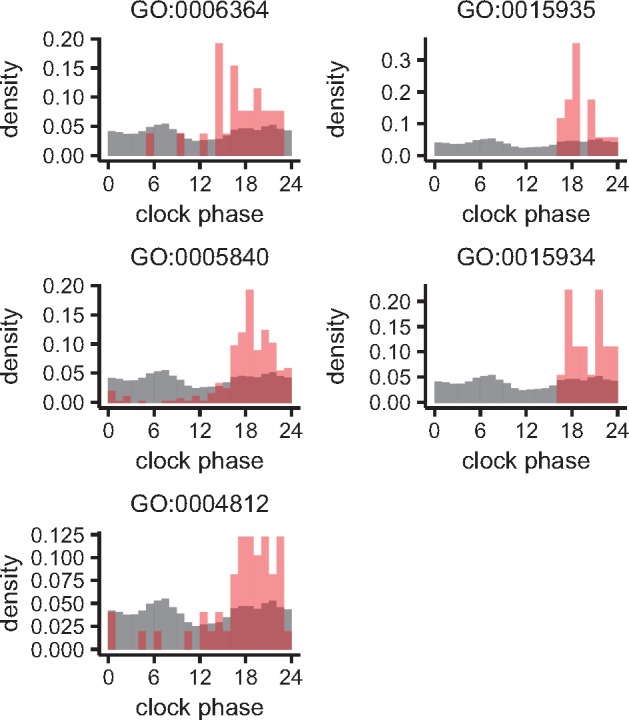
Distributions of clock phases φ of genes annotated with GO:0006364 (rRNA processing), GO:0015935 (small ribosomal subunit), GO:0005840 (ribosome), GO:0015934 (large ribosomal subunit), and GO:0004812 (aminoacyl-tRNA ligase activity), shown as in Figure 5

We investigated the associations between gene annotations and time-of-day characteristics of expressions to assess the consistency between model parameters and biological knowledge. For each gene ontology term with which more than 10 genes are annotated, we compared the distribution of clock phases φ of genes annotated with the term to its background distribution, which is that of genes not annotated with the term. The left-hand panel in Figure 5 indicates that the distribution of φ of genes annotated with protein serine/threonine kinase activity (GO:0004674) was significantly different from its background distribution ($P=2.07\times 10^{-4}$, Watson–Wheeler test, Bonferroni correction). The values of φ of many genes annotated with the term were distributed from before to after midnight. Because there were only four genes annotated with flavonoid biosynthetic process (GO:0009813), we did not perform the statistical test. However, the distribution of φ of those genes was clearly concentrated in the early morning (right-hand panel in Fig. 5). These results are consistent with those of the previous study and a laboratory study of *Arabidopsis* in which the genes implicated in phenylpropanoid biosynthesis showed expressional peaks before subjective dawn (Harmer *et al.*, 2000). Figure 6 shows the distributions of φ of genes annotated with rRNA processing (GO:0006364), small ribosomal subunit (GO:0015935), ribosome (GO:0005840), large ribosomal subunit (GO:0015934), and aminoacyl-tRNA ligase activity (GO:0004812). Although the distribution of genes annotated with rRNA processing (GO:0006364) was not significantly different from its background distribution (*P* = 0.0659; Watson–Wheeler test, Bonferroni correction), the values of φ seem to concentrate in the afternoon. Other distributions (GO:0015935, GO:0005840, GO:0015934, and GO:0004812) were significantly different from their background distributions ($P=5.21\times 10^{-3},\;P=1.01\times 10^{-46}$, *P* =  0.0168, and $P=6.22\times 10^{-6}$, respectively) and the peaks of the distributions were shifted from afternoon to evening in the same order as their biological order in rRNA processing. These results are also consistent with the previous results, which implies that the entrained circadian clock in the field controls the order of the acceleration of translation during the same time period.

We can also use model parameters to form a hypothesis about biological processes occurring in a field. The associations between gene annotations and regression coefficients were also investigated. Before the investigation, we divided the regression coefficients for each gene by the standard deviation of the observed expression of the gene to normalize coefficients. The absolute values of the normalized coefficients of genotype $\beta_{n}^{\left(i\right)}$ for genes annotated with photosynthesis (GO:0015979), thylakoid (GO:0009579), and photosysthetic membrane (GO:0034357) were significantly larger than the background distributions of those values ($P=3.04\times 10^{-6},\;P=5.93\times 10^{-4}$, and $P=1.51\times 10^{-3}$, respectively, Wilcoxon rank sum test, Bonferroni correction). This result implies that the difference in genotype affects photosynthesis. Further, the absolute values of normalized coefficients of the response for genes, for which temperature was selected as an environmental factor, indicated a significant association with phosphorus metabolic process (GO:0006793) ($P=6.93\times 10^{-3}$, Wilcoxon rank sum test, Bonferroni correction). This association suggests that phosphorum metabolism may be affected by fluctuations in temperature.

## 5 Conclusion

In this paper, we proposed a new gene expression model for organisms in a field, based on a previous model (Nagano *et al.*, 2012). The new model vastly reduces the computational time required for parameter optimization and model selection by unifying various types of gate functions and response functions, and introducing group lasso (Yuan and Lin, 2006). By applying the model to synthetically generated RNA-Seq data, we confirmed that the optimized model was consistent with the true gene expression dynamics for most genes. Further, to assess the capability of the new model, we applied it to the same ric plant data that were used in the previous study. The new model offered a comparable or slightly better prediction for most genes.

The model parameters were consistent with those of the previous study and the biological knowledge. This consistency indicates the model’s capability to provide biological insights. In fact, the investigation of model parameters found associations between genotypes and photosynthesis and between the response to temperature and phosphorum metabolism, which were not discovered in molecular biological studies. We can form a hypothesis based on such associations; it will be validated by further experimental studies.

Whereas the previous model was targeted only for microarray data, the new model is applicable to RNA-Seq data. The results of applying the new model to synthetic RNA-Seq data assuming known true models and pseudo RNA-Seq data generated from real microarray data indicated that the model may be useful for the analysis of RNA-Seq data. However, it should be noted that, in this study, the applicability was verified with only simulated data rather than real data. Further verification of performance with real data is required.

In this study, we focused on the time variation of gene expression and analyzed transcriptome data sampled over time. However, the proposed model is applicable to data collected by other sampling strategies, such as multiple treatments at a single time point, only by preparing meteorological data of sufficient length. Further, although we applied the model to the transcriptome data of rice plants in this study, it is applicable to those of other organisms.

The developed package (FIT) offers efficient parameter optimization and model selection. While the parameter optimization and model selection processes for all genes in the previous study required 30 days when a high-performance cluster computer was used, our new package does not incur such a high computational cost. Because the model describes the expression of each gene independently, the parameter optimization and model selection processes can be easily performed in parallel by dividing genes into several groups and performing these processes for each group. For example, it is expected that the parameter optimization and model selection processes can be completed by 10 Amazon EC2 m3.medium instances in half a day. This package will accelerate field transcriptomic studies.

## Funding

This work was supported by Precursory Research for Embryonic Science and Technology (PRESTO), Japan Science and Technology Agency (JST) to A.J.N.; Core Research for Evolutional Science and Technology (CREST), JST to A.J.N.; Accelerated Innovation Research Initiative Turning Top Science and Ideas into High-Impact Values (ACCEL), JST to A.J.N.; and KAKENHI [JP16H06171, JP16H01473 to A.J.N.].

*Conflict of Interest*: none declared.

## Supplementary Material

Supplementary Data
