# Supplementary material for: FIT: statistical modeling tool for transcriptome dynamics under fluctuating field conditions
Source: Bioinformatics. 2017 Jan 31;33(11):1672–80. doi: 10.1093/bioinformatics/btx049 (PMC5447243; doi:10.1093/bioinformatics/btx049)
Supplement: Supplementary Data [file btx049_supp.pdf]

Table S1: Simulated models of variably expressed genes. Here, t denotes the time the sample was taken in units of minutes. Hence, 1440 in the clock term means one day. Temperature at time t is represented by $T(t)$. $\Theta(x)$ is the Heaviside step function, which is 1 if x is larger than 0, otherwise 0. The leftmost column "type" indicates which terms are contained in a model of each gene (c: circadian clock, r: response to temperature, and d: age).

type	expression			
	mean	clock	response to temperature	age
c	α_1	$+2\frac{\cos(2\pi t/24)}{2}$		
	α_2	$+2\frac{\cos(2\pi(t-6)/24)}{2}$		
	α_3	$+2\frac{\cos(2\pi(t-12)/24)}{2}$		
	α_4	$+2\frac{\cos(2\pi(t-18)/24)}{2}$		
r	α_5		$+2\sum_{\tau=1}^{60}\Theta(T(t-\tau/60)-25)/60$	
	α_6		$-2\sum_{\tau=1}^{360}\Theta(T(t-\tau/60)-25)/360$	
	α_7		$+0.4\sum_{\tau=1}^{1440}\max(0,T(t-\tau/60)-25)/1440$	
c+r	α_8	$+\frac{\cos(2\pi t/24)}{2}$	$+\sum_{\tau=1}^{60}\Theta(T(t-\tau/60)-25)/60$	
	α_9	$+\frac{\cos(2\pi(t-6)/24)}{2}$	$+\sum_{\tau=1}^{60}\Theta(T(t-\tau/60)-25)/60$	
	α_{10}	$+\frac{\cos(2\pi(t-12)/24)}{2}$	$-\sum_{\tau=1}^{60}\Theta(T(t-\tau/60)-25)/60$	
	α_{11}	$+\frac{\cos(2\pi(t-18)/24)}{2}$	$-\sum_{\tau=1}^{60}\Theta(T(t-\tau/60)-25)/60$	
	α_{12}	$+\frac{\cos(2\pi t/24)}{2}$	$-\sum_{\tau=1}^{360}\Theta(T(t-\tau/60)-25)/360$	
	α_{13}	$+\frac{\cos(2\pi(t-6)/24)}{2}$	$+\sum_{\tau=1}^{360}\Theta(T(t-\tau/60)-25)/360$	
	α_{14}	$+\frac{\cos(2\pi(t-12)/24)}{2}$	$-\sum_{\tau=1}^{360}\Theta(T(t-\tau/60)-25)/360$	
	α_{15}	$+\frac{\cos(2\pi(t-18)/24)}{2}$	$+\sum_{\tau=1}^{360}\Theta(T(t-\tau/60)-25)/360$	
	α_{16}	$+\frac{\cos(2\pi t/24)}{2}$	$-0.2\sum_{\tau=1}^{1440}\max(0,T(t-\tau/60)-25)/1440$	
	α_{17}	$+\frac{\cos(2\pi(t-6)/24)}{2}$	$+0.2\sum_{\tau=1}^{1440}\max(0,T(t-\tau/60)-25)/1440$	
	α_{18}	$+\frac{\cos(2\pi(t-12)/24)}{2}$	$+0.2\sum_{\tau=1}^{1440}\max(0,T(t-\tau/60)-25)/1440$	
	α_{19}	$+\frac{\cos(2\pi(t-18)/24)}{2}$	$-0.2\sum_{\tau=1}^{1440}\max(0,T(t-\tau/60)-25)/1440$	
c+r+d	α_{20}	$+\frac{\cos(2\pi t/24)}{2}$	$+\sum_{\tau=1}^{60}\Theta(T(t-\tau/60)-25)/60$	$+0.01d$
	α_{21}	$+\frac{\cos(2\pi(t-6)/24)}{2}$	$+\sum_{\tau=1}^{60}\Theta(T(t-\tau/60)-25)/60$	$+0.01d$
	α_{22}	$+\frac{\cos(2\pi(t-12)/24)}{2}$	$-\sum_{\tau=1}^{60}\Theta(T(t-\tau/60)-25)/60$	$+0.01d$
	α_{23}	$+\frac{\cos(2\pi(t-18)/24)}{2}$	$-\sum_{\tau=1}^{60}\Theta(T(t-\tau/60)-25)/60$	$+0.01d$
	α_{24}	$+\frac{\cos(2\pi t/24)}{2}$	$-\sum_{\tau=1}^{360}\Theta(T(t-\tau/60)-25)/360$	$+0.01d$
	α_{25}	$+\frac{\cos(2\pi(t-6)/24)}{2}$	$+\sum_{\tau=1}^{360}\Theta(T(t-\tau/60)-25)/360$	$+0.01d$
	α_{26}	$+\frac{\cos(2\pi(t-12)/24)}{2}$	$-\sum_{\tau=1}^{360}\Theta(T(t-\tau/60)-25)/360$	$-0.01d$
	α_{27}	$+\frac{\cos(2\pi(t-18)/24)}{2}$	$+\sum_{\tau=1}^{360}\Theta(T(t-\tau/60)-25)/360$	$-0.01d$
	α_{28}	$+\frac{\cos(2\pi t/24)}{2}$	$-0.2\sum_{\tau=1}^{1440}\max(0,T(t-\tau/60)-25)/1440$	$-0.01d$
	α_{29}	$+\frac{\cos(2\pi(t-6)/24)}{2}$	$+0.2\sum_{\tau=1}^{1440}\max(0,T(t-\tau/60)-25)/1440$	$-0.01d$
	α_{30}	$+\frac{\cos(2\pi(t-12)/24)}{2}$	$+0.2\sum_{\tau=1}^{1440}\max(0,T(t-\tau/60)-25)/1440$	$-0.01d$
	α_{31}	$+\frac{\cos(2\pi(t-18)/24)}{2}$	$-0.2\sum_{\tau=1}^{1440}\max(0,T(t-\tau/60)-25)/1440$	$-0.01d$

Table S2: Summary of samples collected in 2008.

Start	End	interval	number of samples at each time	total number of samples
7:00 am August 12th	7:00 August 13th	2 h	8	104
10:00 am Jun. 5th	10:00 am Jun. 7th	2 h	1	25
10:00 am Jun. 19th	10:00 am Jun. 21st	2 h	1	25
10:00 am Jul. 3rd	10:00 am Jul. 5th	2 h	1	25
10:00 am Jul. 17th	10:00 am Jul. 19th	2 h	1	25
10:00 am Aug. 7th	10:00 am Aug. 9th	2 h	1	25
10:00 am Aug. 14th	10:00 am Aug. 16th	2 h	1	25
10:00 am Aug. 21st	10:00 am Aug. 23th	2 h	1	25
10:00 am Aug. 28th	10:00 am Aug. 30th	2 h	1	25
10:00 am Sep. 11th	10:00 am Sep. 13th	2 h	1	25
00:00 pm Jun. 3rd	00:00 pm Sep. 23rd	1 week	3	51
00:00 am Jun. 4th	00:00 am Sep. 24th	1 week	2	34
5:00 pm Aug. 7th	8:00 pm Aug 7th	10 min	1	19
3:50 am Aug. 8th	6:00 am Aug. 8th	10 min	2	28

Table S3: Summary of samples collected in 2009.

Start	End	interval	number of samples at each time	total number of samples
00:00 pm Aug. 10th	00:00 pm Aug. 12nd	6 h	2	18
7:00 am Aug. 24th	7:00 am Aug. 25th	2 h	6	78
00:00 pm Aug. 31st	6:00 pm Aug. 31st	6 h	2	4
11:00 am Oct. 8th	11:00 am Oct. 9th	1 day	4	8

Table S4: Fitted coefficients. The leftmost column indicates true models. Cells in boldface indicate non-zero terms in true models.

true type	β_c	β_r	β_d	β_{dc}	β_{dr}	β_n
c	**2.10**	0.00	0.00	0.00	0.00	0
c	**2.00**	0.00	0.00	0.00	0.00	0
c	**2.03**	0.00	0.00	0.00	0.00	0
c	**2.10**	0.00	0.00	0.00	0.00	0
r	0.00	**5.19**	0.00	0.00	0.00	0
r	0.00	**-6.14**	0.00	0.00	0.00	0
r	0.00	**6.91**	0.00	0.00	0.00	0
c+r	**1.01**	**2.87**	0.00	0.00	0.00	0
c+r	**0.99**	**-2.52**	0.00	0.00	0.00	0
c+r	**1.07**	**-3.00**	0.00	0.00	0.00	0
c+r	**1.09**	**-2.65**	0.00	0.00	0.00	0
c+r	**0.84**	**2.71**	0.00	0.00	0.00	0
c+r	**1.00**	**2.75**	0.00	0.00	0.00	0
c+r	**0.98**	**-3.11**	0.00	0.00	0.00	0
c+r	**0.98**	**2.69**	0.00	0.00	0.00	0
c+r	**0.96**	**-8.05**	0.00	0.00	0.00	0
c+r	**0.98**	**4.17**	0.00	0.00	0.00	0
c+r	**1.01**	**3.04**	0.00	0.00	0.00	0
c+r	**1.07**	**-3.92**	0.00	0.00	0.00	0
c+r+d	**0.96**	**2.67**	**0.78**	0.00	0.00	0
c+r+d	**0.00**	**0.00**	**0.00**	0.00	0.00	0
c+r+d	**1.06**	**-2.32**	**0.79**	0.00	0.00	0
c+r+d	**1.02**	**2.64**	**0.90**	0.00	0.00	0
c+r+d	**0.99**	**-3.01**	**0.83**	0.00	0.00	0
c+r+d	**0.93**	**3.07**	**0.82**	0.00	0.00	0
c+r+d	**1.00**	**-3.12**	**-0.94**	0.00	0.00	0
c+r+d	**1.17**	**2.92**	**-0.83**	0.00	0.00	0
c+r+d	**1.03**	**-6.54**	**-0.86**	0.00	0.00	0
c+r+d	**1.08**	**0.00**	**-0.55**	0.00	0.00	0
c+r+d	**1.01**	**4.25**	**-0.78**	0.00	0.00	0
c+r+d	**0.98**	**-3.39**	**-0.88**	0.00	0.00	0

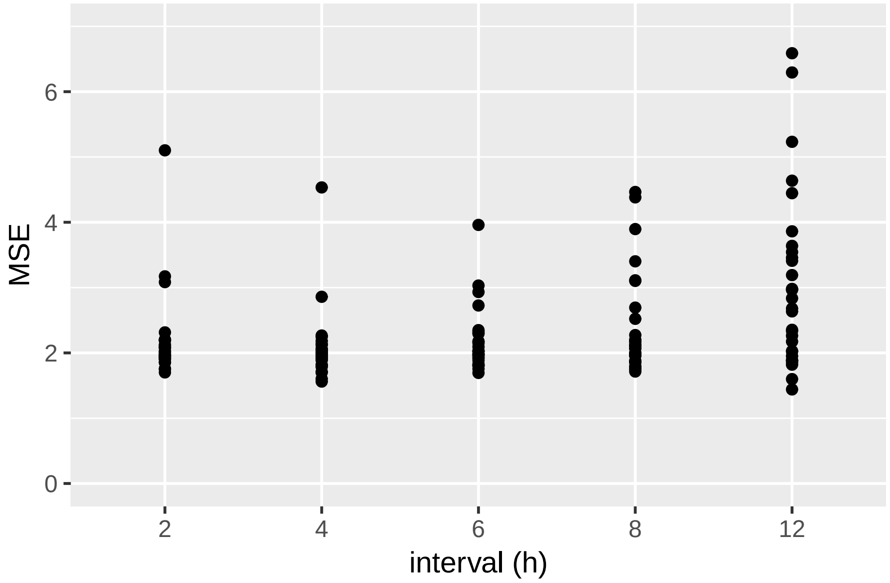

Figure S1: Mean squared errors (MSE) of predicted values from synthetic values of gene expressions for all sampling types. The x axis indicates intervals of sampling. When the interval is 6 h, there were 2 genes, for which parameter optimization failed and mean squared errors exceeded the y axis range. Hence, these two points were not plotted.

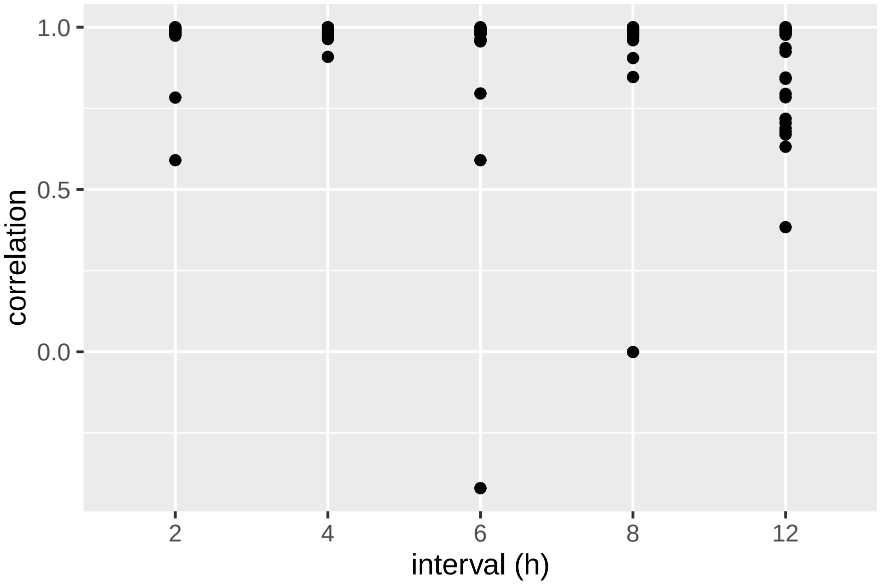

Figure S2: Correlation coefficients between predicted and synthetic values of gene expressions for all sampling types.

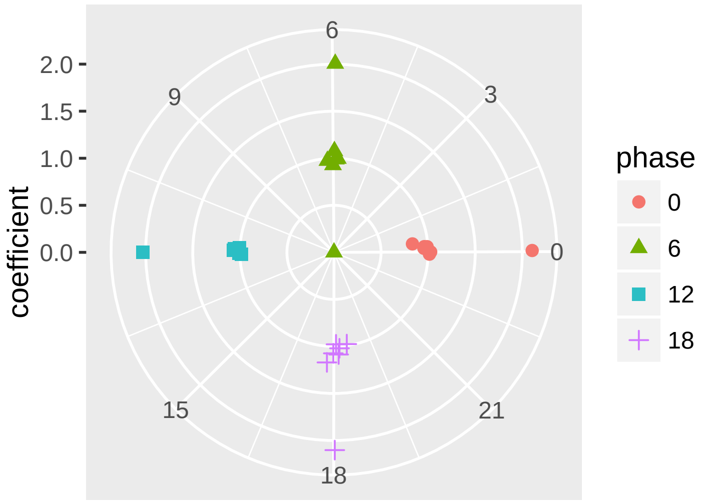

Figure S3: Polar plot of circadian clocks. Phases and coefficients are indicated by angle and radius, respectively. Phases of the true models are indicated by shapes and colors of markers.

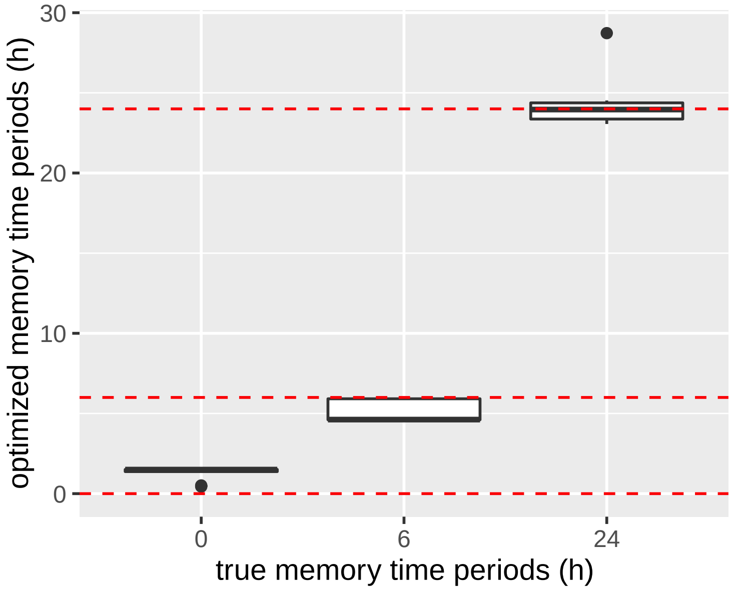

Figure S4: Boxplot of optimized memory time periods $p^{\cdot(i)}$. The horizontal axis and horizontal red dashed lines indicate memory time periods of the true models.

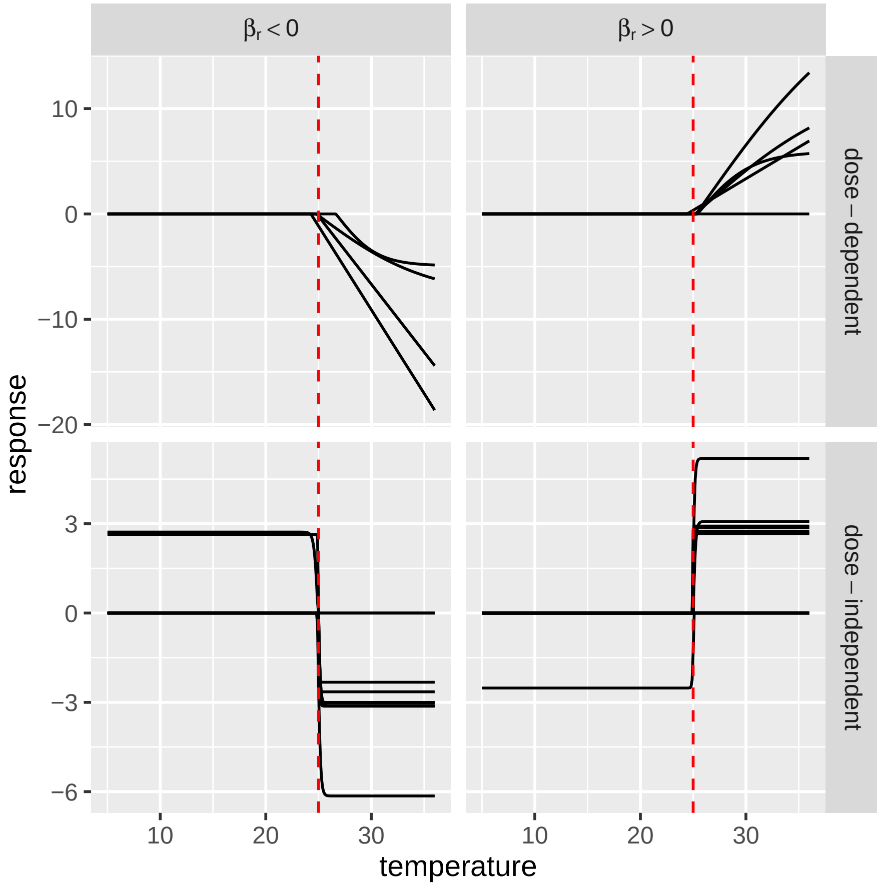

Figure S5: Plots of responses to temperature classified by signs of coefficients and dose-dependencies of the true models. The red dashed lines indicate the true threshold (25°C)
